# Slit2N and Robo4 regulate lymphangiogenesis through the VEGF-C/VEGFR-3 pathway

**DOI:** 10.1186/1478-811X-12-25

**Published:** 2014-04-07

**Authors:** Jinlong Yu, Xuefeng Zhang, Paula M Kuzontkoski, Shuxian Jiang, Weiquan Zhu, Dean Y Li, Jerome E Groopman

**Affiliations:** 1 Division of Experimental Medicine, Beth Israel Deaconess Medical Center, Harvard Medical School, 330 Brookline Ave, Boston, MA 02115, USA; 2 Department of Molecular Medicine and Molecular Medicine Program, University of Utah, Salt Lake City, UT 84112, USA; 3 The Key Laboratory for Human Disease Gene Study of Sichuan Province, Institute of Laboratory Medicine, Sichuan Academy of Medical Sciences & Sichuan Provincial People’s Hospital, Chengdu, Sichuan 610072, China; 4 Current Address: Division of Hematology/Oncology, Department of Medicine, University of Florida, Gainesville, FL 32610, USA

**Keywords:** Slit2, Robo4, VEGF-C, VEGFR-3, Akt, PI3K, Proliferation, Migration, Lymphangiogenesis

## Abstract

**Background:**

Signaling through vascular endothelial growth factor C (VEGF–C) and VEGF receptor 3 (VEGFR-3) plays a central role in lymphangiogenesis and the metastasis of several cancers via the lymphatics. Recently, the Slit2/Robo4 pathway has been recognized as a modulator of vascular permeability and integrity. Signaling via the Robo receptor inhibits VEGF-mediated effects; however, its effects on lymphatic endothelial cell function have not been well characterized.

**Results:**

We found that pretreatment with Slit2N, an active fragment of Slit2, inhibited VEGF-C-mediated lung-derived lymphatic endothelial cell (L-LEC) proliferation, migration, and *in vitro* tube formation. Slit2N induced the internalization of VEGFR-3, which blocked its activation, and inhibited the activation of the PI3K/Akt pathway by VEGF-C in L-LECs. Moreover, we found that inhibition of VEGF-C-induced effects by Slit2N was Robo4-dependent.

**Conclusion:**

These results indicate that Slit2N/Robo4 modulates several key cellular functions, which contribute to lymphangiogenesis, and identify this ligand-receptor pair as a potential therapeutic target to inhibit lymphatic metastasis of VEGF-C-overexpressing cancers and manage lymphatic dysfunctions characterized by VEGF-C/VEGFR-3 activation.

## Lay abstract

Cellular signaling initiated by the binding of vascular endothelial growth factor C (VEGF-C) to the receptor, VEGFR-3, is central to the growth of lymphatic channels, their constituent endothelial cells, and the spread of several types of cancer via the lymphatic system. Signaling through the ligand-receptor pair, Slit2/Robo4, modulates the permeability and integrity of vascular endothelium, the cells that line blood vessels. The Slit2/Robo pathway inhibits cellular effects induced by VEGF; however, its effects on lymphatic channels and lymphatic endothelium have not been fully studied.

We found that pretreating lung-derived lymphatic endothelial cells (L-LECs) with Slit2N, an active fragment of the protein Slit2, inhibited VEGF-C-induced functions critical for the formation of the lymphatics, i.e. lymphangiogenesis. Specifically, it blocked the growth and migration of L-LECs, and the formation of tube-like structures on a gelatinous matrix. Slit2N induced the internalization VEGR-3, which blocked its activation, and inhibited signaling through PI3K/Akt, a pathway that modulates diverse cellular processes, including cell growth and cancer progression. In addition, we found that inhibition of VEGF-C-induced effects by Slit2N required sufficient levels of Robo4, indicating that Robo4 is the receptor to which Slit2N binds to inhibit the aforementioned effects.

These results indicate that Slit2N/Robo4 modulates key cellular functions that contribute to lymphangiogenesis, and identify this ligand-receptor pair as a potential drug target to inhibit cancer metastasis via the lymphatic system and to treat other lymphatic pathologies characterized by abnormal VEGF-C/VEGFR-3 signaling.

## Background

The lymphatic system plays critical roles in the maintenance of fluid homeostasis, immune response, and tumor metastasis [1]. VEGF-C/VEGFR-3 signaling is a key modulator of this system [2-4]. Many cancers express VEGF-C [5-16]. Clinical studies demonstrate that VEGF-C levels correlate with lymph node metastasis and poor prognosis [8,14,17-25], and multiple tumor types preferentially metastasize through lymphatic vessels versus blood vascular dissemination or direct seeding [26,27]. Tumor-induced lymphangiogenesis plays an active role in the induction of metastasis to the lymph nodes in these cancers [28].

Given the role of the VEGF-C/VEGFR-3 signaling in tumor lymphangiogenesis and metastasis, inhibiting this pathway with soluble VEGFR-3, neutralizing antibodies to VEGFR-3 or VEGF-C, or suppressing VEGF-C expression with siRNAs can reduce lymph node and organ metastasis in rodent models [29-32]. Moreover, VEGF-C/VEGFR-3 signaling does not appear to be required for the maintenance of lymphatic vessels beyond development, since prolonged inhibition of VEGFR-3 signaling in animals impedes lymph node metastasis with no apparent effects on preexisting, mature, lymphatic vessels in adjacent tissue [31,33-35]. These data suggest that VEGF-C/VEGFR-3 signaling in lymphatic endothelium may be an attractive target to restrict cancer metastasis via the lymphatics [36].

Tumor cells and tumor-associated macrophages appear to be the primary sources of lymphangiogenic factors including VEGF-C [37-39]. Its receptor, VEGFR-3, is predominantly expressed by lymphatic endothelial cells [40]. Of note, in mouse models of melanoma and breast cancer, lymphangiogenesis in the draining lymph nodes precedes the arrival of any tumor cells, and is enhanced further after metastasis to the nodes [7,41]. These data suggest that tumor-secreted VEGF-C may act in a paracrine fashion by draining to the sentinel lymph nodes and modulating the lymphatic microenvironment both before metastasis and after tumor cells have migrated to the lymph nodes [41]. It is this model of the effects of exogenous VEGF-C on LECs that we recapitulate here.

The Slit and Robo proteins were first characterized as modulators of axon guidance and repulsion in central nervous system development [42]. The Slit proteins (Slits 1–3) are a group of glycoproteins containing various functional domains including a leucine-rich repeat (LRR) region, which is important for binding to their cognate Robo receptors [43]. Among the four Robo proteins that have been identified in mammals [44-47], the structure of Robo4 is unique. In contrast to the other Robo proteins, its extracellular region contains fewer Ig-like domains and fewer fibronectin (Fn) type III repeats; and its intracellular domain contains only two of five cytoplasmic conserved (CC) motifs [48]. Its nearly exclusive expression on endothelial cells also distinguishes Robo4 from the other Robo proteins [48]. *In vivo*, Slit2 is proteolytically cleaved into an N-terminal, 140 kDa fragment (Slit2N), and a C-terminal, 55–60 kDa fragment (Slit2C) [42,49]. Importantly, this cleavage was also observed in kidney cells manipulated to overexpress recombinant Slit2, demonstrating that the proteolytic processing of Slit2 is recapitulated *in vitro*[42]. Functional studies have shown that Slit2N, but not Slit2C, can associate with the Robos and modulate signaling downstream of these receptors [49-51]; therefore, we employed recombinant Slit2N in this study.

In addition to their roles in nervous system development, Slit and Robo proteins modulate vascular endothelial cell function. Slit2/Robo4 inhibits *in vitro* transwell migration of human microvascular endothelial cells (HMVEC) and human umbilical vein endothelial cells (HUVEC); they promote vascular stability and inhibit hyperpermeability in a mouse model of retinal permeability [52]. Accumulating evidence indicates that Slit2/Robo4 modulates angiogenesis by inhibiting VEGF signaling in vascular endothelial cells [53,54]; in contrast, several studies have reported that Slit2/Robo activation can stimulate vascular endothelial cell proliferation and migration, and increase tumor metastasis [55-57]. Remarkably, little is known about the effects of Slit/Robo on the lymphatic system; therefore, we sought to evaluate signaling via Slit2N/Robo4 on VEGF-C-induced functions in L-LECs. We observed that Slit2N and Robo4 inhibited VEGF-C-induced lymphatic endothelial cell proliferation, migration, and tube formation by blocking the activation of VEGFR-3 and signaling through Phosphatidylinositide 3-kinase (PI3K) and Protein kinase B (Akt). In addition, Slit2N/Robo4 enhanced the internalization of VEGFR-3. As novel mediators of lymphangiogenesis, Slit2 and Robo4 may be attractive targets for therapy aimed at controlling tumors with enhanced VEGF-C/VEGFR-3 signaling that metastasize through the lymphatic system, and lymphatic pathology induced by signaling through this pathway.

## Results

### Slit2N inhibits VEGF-C-enhanced growth, migration, and tube formation of L-LECs

**Figure 1:**
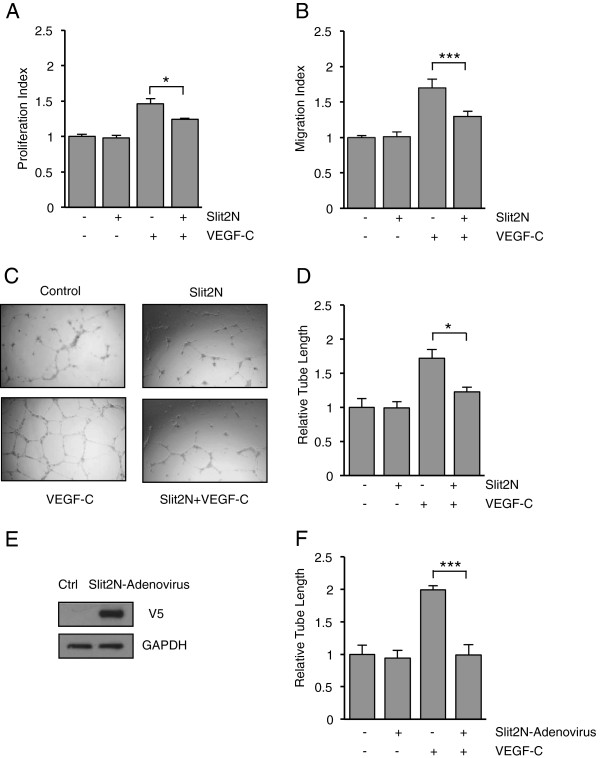
**Slit2N inhibits VEGF-C-enhanced growth, migration and tube formation of L-LECs. (A)** Proliferation of L-LECs as assessed by MTS assay after incubation with control, 10 nM Slit2N, or VEGF-C [100 ng/ml]; or after preincubation with Slit2N, then VEGF-C. Data represent the mean ± SD of 3 independent experiments (*p < 0.05). **(B)** Transwell migration of L-LECs after treatment with control, 10 nM Slit2N, or VEGF-C [100 ng/ml]; or after preincubation with Slit2N, then VEGF-C. Data represent the mean ± SD of 3 independent experiments (***p < 0.001). **(C)** Representative tube formation assay on ECM of L-LECs after treatment with control, 10 nM Slit2N, or VEGF-C [100 ng/ml]; or after preincubation with Slit2N, then VEGF-C. **(D)** Average length of L-LEC tubes as assessed by *in vitro* tube formation assay on ECM after treatment with 10 nM Slit2N, VEGF-C [100 ng/ml]; or after preincubation with Slit2N, then VEGF-C, relative to average tube length of untreated cells (Slit2N “-”, VEGF-C “-”). Data represent the mean ± SD of 3 independent experiments (*p < 0.05). **(E)** Representative Western blot analysis of Slit2N expression in L-LECs transduced with a control adenovirus (Ctrl) or with an adenovirus expressing V5-tagged Slit2N. GAPDH used as loading control. **(F)** Average length of L-LEC tubes in L-LECs transduced with a control adenovirus (Slit2N-Adenovirus “-”) or in L-LECs transduced with a Slit2N-expressing adenovirus (Slit2N-Adenovirus “+”) as assessed by *in vitro* tube formation assay on ECM after incubation with control (VEGF-C “-”) or with VEGF-C [100 ng/ml] (VEGF-C “+”), relative to average tube length of untreated L-LECs transduced with control adenovirus (Slit2N-Adenovirus “-”, VEGF-C “-”). Data represent the mean ± SD of 3 independent experiments (***p < 0.001).

To evaluate the effects of Slit/Robo on VEGF-C-modulated lymphatic endothelial cell functions, we incubated L-LECs with PBS, Slit2N, VEGF-C, or Slit2N followed by VEGF-C, and assessed proliferation and migration (Figure 1A and B, respectively). We observed that Slit2N alone had no significant effect on the proliferation of L-LECs; however, 100 ng/ml VEGF-C greatly enhanced their growth, and pretreatment with Slit2N significantly inhibited this VEGF-C-enhanced proliferation (Figure 1A).

In a transwell migration assay, Slit2N had no discernible effect on the migration of L-LECs, however, 100 ng/ml VEGF-C significantly enhanced migration, and pretreatment with Slit2N inhibited this VEGF-C-enhanced transwell migration (Figure 1B).

As an *in vitro* correlate for lymphangiogenesis [58], we also assessed the ability of L-LECs to form 2-dimensional, endothelial cell enclosures on an artificial extracellular matrix (Figure 1C). While Slit2N alone had no effect on the average length of L-LEC tubes, average length increased significantly after 100 ng/ml VEGF-C treatment (Figure 1D). Pretreatment with recombinant Slit2N inhibited this VEGF-C-induced tube length enhancement (Figure 1D).

To confirm the inhibitory effect of Slit2N on VEGF-C-induced tube formation, we transduced a Slit2N-expressing adenovirus or a control virus into L-LECs, and observed Slit2N expression by Western blot analysis, 24 hours post-transduction (Figure 1E). Subsequently, we transduced L-LECs with the Slit2N adenovirus (Slit2N-Adenovirus “+”) or control (Slit2N-Adenovirus “-”), and examined tube formation after VEGF-C stimulation (Figure 1F). Both sets of untreated L-LEC transductants (VEGF-C “-” lanes) formed relatively short tubes (Figure 1F). While VEGF-C significantly enhanced tube length in the control-transduced L-LECs, VEGF-C did not in the L-LECs transduced with Slit2N adenovirus (Figure 1F). This is consistent with the effects of recombinant Slit2N (Figure 1D). Taken together, these data indicate that Slit2N can inhibit VEGF-C-enhanced growth, migration and tube formation in L-LECs, and suggest that Slit2N may inhibit VEGF-C-induced lymphangiogenesis *in vivo*.

Studies of VEGF-C *in vitro* are routinely conducted at concentrations between 10 ng/ml and 1000 ng/ml [59-62]. We determined that [100 ng/ml] was the lowest concentration at which VEGF-C had a significant functional effect on L-LECs (data not shown); therefore, we conducted our studies at this final concentration.

### Slit2N reduces VEGF-C-induced activation of VEGFR-3

**Figure 2:**
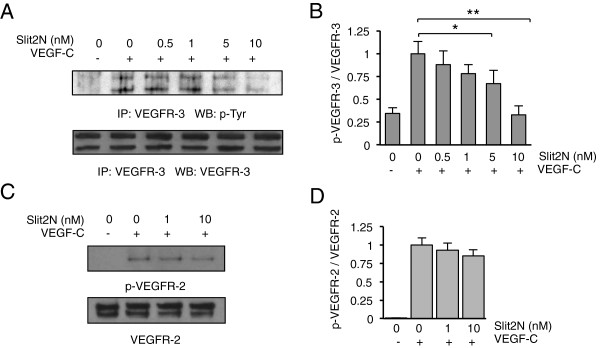
**Slit2N attenuates VEGF-C-induced activation of VEGFR-3 in L-LECs. (A)** Representative VEGFR-3 IP/Western blot analysis of phosphorylated VEGFR-3 in L-LECs after pretreatment with various concentrations of Slit2N, and incubation with VEGF-C [100 ng/ml]. Total VEGFR-3 used as loading control. **(B)** Quantitative analysis of VEGFR-3 IP/Western blot analysis of phosphorylated VEGFR-3 in L-LECs after pretreatment with various concentrations of Slit2N, and incubation with VEGF-C. Band intensities from Figure 2A were determined by densitometry. The ratio of p-VEGFR-3/total VEGFR-3 of L-LECs treated with VEGF-C alone (2^nd^ lanes from the left) was set to “1” and the ratios of all other conditions were calculated vs. this experimental condition. Data represent the mean ± SD of 3 independent experiments (*p < 0.05; **p < 0.01). **(C)** Representative Western blot analysis of phosphorylated VEGFR-2 in L-LECs after pretreatment with various concentrations of Slit2N, and incubation with VEGF-C [100 ng/ml]. Total VEGFR-2 used as loading control. **(D)** Quantitative analysis of Western blot analysis of phosphorylated VEGFR-2 in L-LECs after pretreatment with various concentrations of Slit2N, and incubation with VEGF-C. Band intensities from Figure 2C were determined by densitometry. The ratio of p-VEGFR-2/total VEGFR-2 of L-LECs treated with VEGF-C alone (2^nd^ lanes from the left) was set to “1” and the ratios of all other conditions were calculated vs. this experimental condition. Data represent the mean ± SD of 3 independent experiments.

To promote lymphangiogenesis, VEGF-C binds to VEGFR-2 and VEGFR-3, which induces their dimerization, activation, and the initiation of intracellular signaling [2,63]. We examined the phosphorylation of VEGFR-2 and VEGFR-3 in L-LECs after incubation with PBS, VEGF-C, or Slit2N followed by VEGF-C, and observed low, basal phosphorylation of the VEGFR-3 isoforms in control-treated L-LECs (Figure 2A and B). Incubation with VEGF-C significantly enhanced the activation of these isoforms, and Slit2N inhibited this VEGF-C-enhanced activation in a dose-dependent manner (Figure 2A and B). Similarly, VEGF-C activated VEGFR-2; however, Slit2N did not significantly inhibit VEGF-C-induced VEGFR-2 activation (Figure 2C and D). These data indicate that Slit2N predominantly affects L-LEC proliferation, migration, and tube formation by modulating signaling through VEGF-C and VEGFR-3.

### The effects of Slit2N and VEGF-C on total VEGFR-3 levels and on VEGFR-3 surface presentation/internalization in L-LECs

The activity of VEGFR-3 is modulated through a variety of mechanisms, including its association with VEGFR-2 and alpha 5 integrin [64,65]. Using VEGFR-3 immunoprecipitation and Western blot analysis, we examined whether Slit2N disrupted the interaction between VEGFR-3 and either of the aforementioned molecules. There was no discernible basal interaction between VEGFR-3 and alpha 5 integrin, a very low basal interaction between VEGFR-3 and VEGFR-2 in untreated L-LECs, and Slit2N had no effect on these associations (Additional file 1); therefore, we examined another potential mechanism by which Slit2N might inhibit VEGF-C-induced activation of VEGFR-3, VEGFR-3 internalization [2].

**Figure 3:**
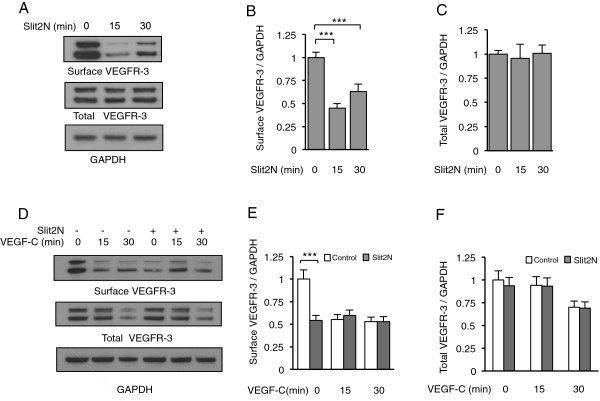
**Effects of Slit2N and VEGF-C on total VEGFR-3 levels and VEGFR-3 surface presentation/internalization in L-LECs. (A)** Representative Western blot analysis of surface and total VEGFR-3 in L-LECs after incubation with 10 nM Slit2N for indicated times. GAPDH: loading control. **(B)** Quantitative analysis of surface VEGFR-3 in L-LECs after incubation with 10 nM Slit2N for indicated times. Band intensities from Figure 3A were determined by densitometry. Ratio of surface VEGFR-3/GAPDH in untreated L-LECs (“0” lanes) was set to “1” and values of other ratios were calculated vs. this control. **(C)** Quantitative analysis of total VEGFR-3 in L-LECs after incubation with 10 nM Slit2N for indicated times. Band intensities from Figure 3A were determined by densitometry. Ratio of total VEGFR-3/GAPDH in untreated L-LECs (“0” lanes) was set to “1” and values of other ratios were calculated vs. untreated control. **(D)** Representative Western blot analysis of membrane-bound VEGFR-3 (Surface VEGFR-3) and total VEGFR-3 in L-LECs after incubation with VEGF-C alone [100 ng/ml], and 10 nM Slit2N pretreatment + VEGF-C for indicated times. GAPDH: loading control. **(E)** Quantitative analysis of surface VEGFR-3 in L-LECs after incubation with VEGF-C alone [100 ng/ml] (Control), and 10 nM Slit2N pretreatment + VEGF-C for indicated times. Band intensities from Figure 3D were determined by densitometry. Ratio of surface VEGFR-3/GAPDH of untreated L-LECs was set to “1” and values of all other ratios were calculated vs. untreated control. **(F)** Quantitative analysis of total VEGFR-3 in L-LECs after incubation with VEGF-C alone [100 ng/ml] (Control), and 10 nM Slit2N pretreatment + VEGF-C for indicated times. Band intensities from Figure 3D were determined by densitometry. Ratio of total VEGFR-3/GAPDH of untreated L-LECs was set to “1” and values of other ratios were calculated vs. untreated control. Data for **(B)**, **(C)**, **(E)** and **(F)** represent the mean ± SD of 3 independent experiments (***p < 0.001).

We incubated L-LECs with recombinant Slit2N for 0, 15, and 30 minutes, labeled cell surface proteins with biotin, lysed the cells to generate total cell lysates, and isolated the membrane fraction with streptavidin immunoprecipitation. By Western blot analysis we examined the effect of Slit2N on VEGFR-3 levels in the membrane fraction and total cell lysates. There was no change in overall VEGFR-3 expression in the total cell lysates (Figure 3A and C); however, after 15 minutes, Slit2N decreased surface VEGFR-3 expression by more than 50% (Figure 3A and B). After 30 minutes, there was a small increase in the amount of VEGFR-3 presented on the cell surface as compared to levels at 15 minutes (Figure 3A and B). These data indicate that Slit2N induces the internalization of VEGFR-3, but does not affect total VEGFR-3 levels in L-LECs.

To examine the effects of VEGF-C on VEGFR-3, and the effects of Slit2N on VEGF-C-induced modulation of VEGFR-3, we incubated L-LECs with VEGF-C for 0,15, and 30 minutes, or we pretreated L-LECs for 1 hour with Slit2N, then incubated them with VEGF-C; lysed the cells, and isolated the membrane fraction from the total cell lysates as described above. By Western blot analysis we examined VEGFR-3 levels in the membrane fraction and total cell lysates. VEGF-C alone decreased cell surface VEGFR-3 by about 50% after 15 and 30 minutes incubation. After 15 minutes, total VEGFR-3 levels were unchanged, but dropped by about 25% after 30 minutes (Figure 3D, E, and F). Surface expression of VEGFR-3 and total VEGFR-3 in L-LECs incubated with VEGF-C alone for 15 and 30 minutes was nearly identical to L-LECs pretreated with Slit2N, and then incubated with VEGF-C for 15 minutes and 30 minutes (Figure 3D, E, and F). These data indicate that Slit2N has little or no effect on VEGF-C-induced modulation of VEGFR-3.

### Slit2N inhibits VEGF-C-induced PI3K/Akt activity

**Figure 4:**
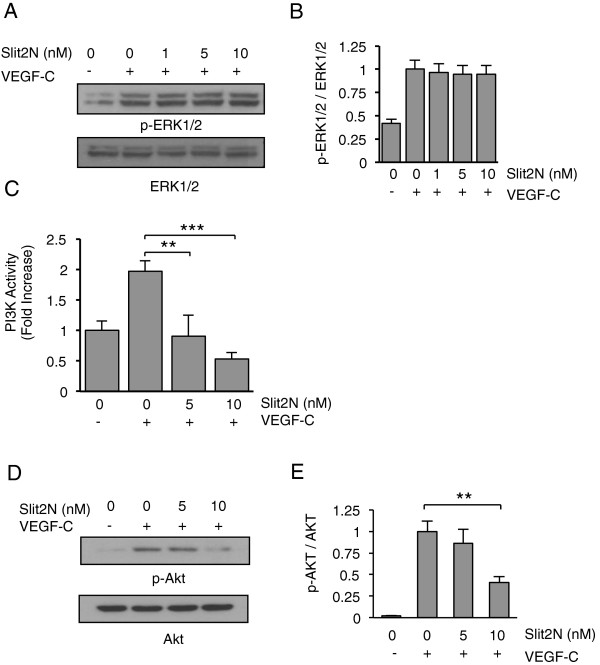
**Slit2N inhibits VEGF-C-enhanced PI3K activity and Akt phosphorylation in L-LECs. (A)** Representative Western blot analysis of phosphorylated ERK1/2 in L-LECs, after treatment with control, VEGF-C alone [100 ng/ml]; or after preincubation with various concentrations of Slit2N, then VEGF-C. Total ERK1/2 used as loading control. **(B)** Quantitative analysis of phosphorylated ERK1/2 in L-LECs, after treatment with control, VEGF-C alone [100 ng/ml]; or after preincubation with various concentrations of Slit2N, then VEGF-C. Band intensity of each lane from Figure 4A was determined by densitometry. The ratio of p-ERK1/2 to total ERK1/2 of L-LECs incubated with VEGF-C alone was set to “1” and values of all other ratios were calculated vs. this control. Data represent the mean ± SD of 3 independent experiments. **(C)** PI3K activity by ELISA in L-LECs incubated with various concentrations of Slit2N and/or VEGF-C [100 ng/ml]. Data represent the mean ± SD of 3 independent experiments (**p < 0.01, ***p < 0.001). **(D)** Representative Western blot analysis of phosphorylated Akt in L-LECs incubated with a control, VEGF-C [100 ng/ml]; or preincubated with various concentrations of Slit2N, then VEGF-C. Total Akt used as loading control. **(E)** Quantitative analysis of phosphorylated Akt in L-LECs, after treatment with control, VEGF-C alone [100 ng/ml]; or after preincubation with various concentrations of Slit2N, then VEGF-C. Band intensity of each lane from Figure 4D was determined by densitometry. The ratio of p-Akt to total Akt of L-LECs incubated with VEGF-C alone was set to “1” and values of all other ratios were calculated vs. this control. Data represent the mean ± SD of 3 independent experiments (**p < 0.01).

To determine which VEGF-C/VEGFR-3 signaling pathways are affected by Slit2N in L-LECs, we examined the activation of ERK1/2, a key downstream molecule in the VEGF-C/VEGFR-3 signaling cascade, and PI3K activity, which can also be enhanced by signaling through VEGF-C/VEGFR-3 [59]. We found that neither Slit2N alone (Additional file 2), nor Slit2N pretreatment before VEGF-C incubation, affected the phosphorylation of ERK1/2 in L-LECs (Figure 4A and B); however, PI3K activity increased significantly after treatment with VEGF-C, and pretreatment with 5 nM and 10 nM Slit2N completely inhibited this VEGF-C-enhanced activity (Figure 4C). We also examined the effects of VEGF-C and Slit2N on the activation of the PI3K downstream signaling molecule, Akt. We found that Slit2N alone had no effect on Akt activation (Additional file 3); however, VEGF-C significantly enhanced Akt phosphorylation in L-LECs, and pretreatment with Slit2N decreased its activation in a dose-dependent manner (Figure 4D and E). These data indicate that Slit2N inhibits VEGF-C-induced activation of PI3K/Akt, but not VEGF-C-induced activation of ERK1/2. The differences in the effects of Slit2N on these VEGF-C/VEGFR-3 effectors suggest that there are likely other signaling molecules in these pathways that are differentially modulated by Slit2N.

### Robo4 is required for Slit2N to inhibit the activation of VEGFR-3 by VEGF-C

**Figure 5:**
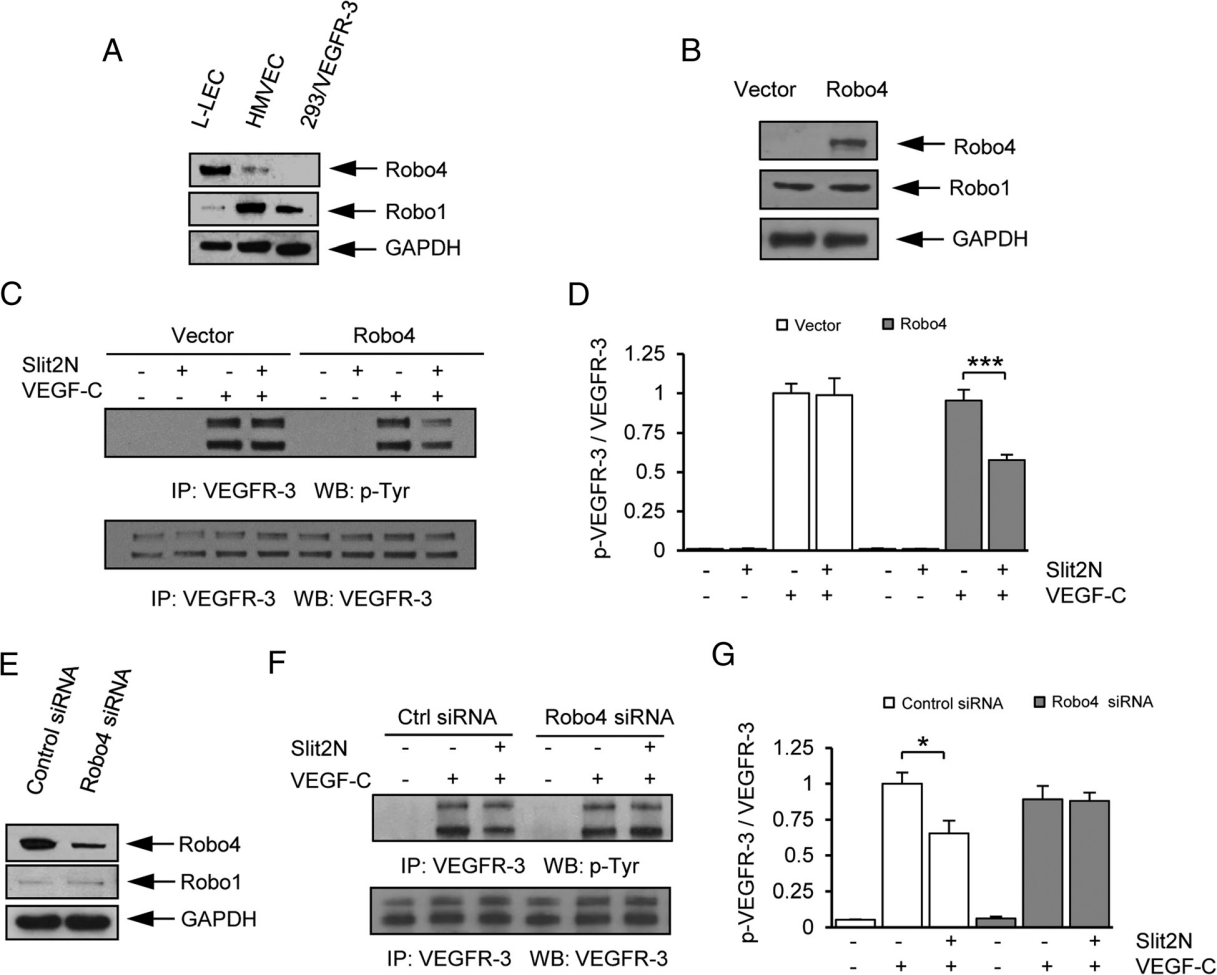
**Slit2N inhibits VEGF-C-induced activation of VEGFR-3 in 293/VEGFR-3 transfectants and L-LECs in a Robo4-dependent manner. (A)** Representative Western blot analysis of Robo1 and Robo4 expression in L-LECs, HMVECs, and 293/VEGFR-3 cells. GAPDH used as loading control. **(B)** Representative Western blot analysis of Robo4 and Robo1 expression in 293/VEGFR-3 cells, 24 h after transfection with pCMV-RFP (vector) or pCMV-RFP-Robo4 (Robo4). GAPDH used as loading control. **(C)** Representative VEGFR-3 IP/Western blot analysis of phosphorylated VEGFR-3. 293/VEGFR-3 cells were transfected with pCMV-RFP (vector) or pCMV-RFP-Robo4 (Robo4). After 48 h, cells were incubated with a control (“- -”), 10 nM Slit2N, or VEGF-C [100 ng/ml]; or pretreated with Slit2N, then VEGF-C. Total VEGFR-3 used as loading control. **(D)** Quantitative analysis by densitometry of Figure 5C. The ratio of p-VEGFR-3/VEGFR-3 in vector-transfected L-LECs incubated with VEGF-C alone was set to “1” and all other ratios were determined vs. this control. Data represent the mean ± SD of 3 independent experiments (***p < 0.001). **(E)** Representative Western blot analysis of Robo4 and Robo1 expression in L-LECs, 24 h after transfection with control siRNAs or Robo4-specific siRNAs. GAPDH used as loading control. **(F)** Representative VEGFR-3 IP/Western blot analysis of phosphorylated VEGFR-3 in L-LECs transfected with control siRNAs or Robo4-specific siRNAs, after incubation with control, VEGF-C [100 ng/ml]; or pretreatment with 10 nM Slit2N, then VEGF-C. Total VEGFR-3 used as loading control. **(G)** Quantitative analysis by densitometry of Figure 5F. The ratio of p-VEGFR-3/VEGFR-3 in control siRNA-transfected L-LECs incubated with VEGF-C alone was set to “1” and all other ratios were determined vs. this control. Data represent the mean ± SD of 3 independent experiments (*p < 0.05).

Among the Slit receptors, Robo4 is expressed almost exclusively in proliferating endothelium and tumor endothelium [45,66]; however, a recent study has also demonstrated Robo1 expression in human lymphatic endothelial cells and its interaction with Slit2 [56]; therefore, by Western blot analysis, we compared the levels of Robo4 and Robo1 in primary L-LECs, primary dermal HMVECs, and 293/VEGFR-3, human embryonic kidney cells manipulated to express VEGFR-3 (as a non-endothelial control). All three cell types expressed Robo1: highest in the HMVECs and lowest in the L-LECs (Figure 5A). Both types of primary endothelial cells expressed Robo4; however, its expression was much higher in L-LECs as compared to HMVECs (Figure 5A). Consistent with its nearly exclusive endothelial expression, we detected no Robo4 in the 293/VEGFR-3 cells (Figure 5A). Since L-LECs expressed the highest comparative levels of Robo4 and the lowest comparative levels of Robo1, we focused on the potential effects of Robo4 on Slit2N-mediated inhibition of VEGF-C/VEGFR-3 signaling in these cells.

We transiently transfected a red fluorescent protein (RFP)-tagged Robo4 expression plasmid or a vector control into the 293/VEGFR-3 cells. By Western blot analysis, we confirmed that while Robo4 was not expressed in the control-transfected cells, it was expressed in the cells transfected with the Robo4 plasmid; and Robo1 expression was identical in both sets of transfectants (Figure 5B). We incubated these cells with PBS, Slit2N, VEGF-C, or Slit2N followed by VEGF-C, immunoprecipitated with VEGFR-3, and examined the phosphorylation of the VEGFR-3 isotypes by Western blot analysis, using a phospho-tyrosine antibody (Figure 5C). Activated VEGFR-3 was not expressed in the untreated 293/VEGFR-3 transients transfected with either the control plasmid or the Robo4-RFP plasmid, and incubation with Slit2N alone had no effect on VEGFR-3 phosphorylation in either group of transfectants (Figure 5C and D, Vector +/− and Robo4 +/−). VEGF-C induced VEGFR-3 activation in both groups of transfectants (Figure 5C and D, Vector −/+ and Robo4 −/+). Treatment with Slit2N before VEGF-C incubation had no effect on activation of the VEGFR-3 isoforms in the vector control group (Figure 5C and D, Vector +/+); however, Slit2N inhibited VEGFR-3 activation in the Robo4-expressing transfectants (Figure 5C and D, Robo4 +/+). These data indicate that Robo4 expression is not required for VEGF-C-induced activation of VEGFR-3 in 293/VEGFR-3 cells; however, sufficient levels of Robo4 are required for Slit2N-modulated inhibition of VEGF-C-induced VEGFR-3 activation.

To extend this finding to lymphatic endothelial cells, we transiently transfected L-LECs with control siRNAs or Robo4-specific siRNAs. After 24 hours, we confirmed a reduction in Robo4 expression by Western blot analysis, and a low, unchanged Robo1 expression in both sets of transfectants (Figure 5E). We incubated these transfectants with PBS, Slit2N, VEGF-C, or Slit2N followed by VEGF-C, immunoprecipitated with VEGFR-3, and examined VEGFR-3 phosphorylation by immunoblot, as above. We observed no VEGFR-3 activation in either group of untreated L-LEC transfectants (Figure 5F and G, Control siRNA −/− and Robo4 siRNA −/−) and VEGF-C induced the phosphorylation of VEGFR-3 in both groups of transfectants (Figure 5F and G, Control siRNA −/+ and Robo4 siRNA −/+). Pretreatment with Slit2N inhibited the VEGF-C-induced activation of VEGFR-3 in the L-LECs with endogenous Robo4 expression (Figure 5F and G, Control siRNA +/+); however, it had no discernible effect on VEGFR-3 activation in the L-LECs with reduced levels of Robo4 (Figure 5F and G, Robo4 siRNA +/+). These data indicate that Robo4 is not required for VEGF-C-induced activation of VEGFR-3 in L-LECs; however, adequate expression of Robo4 is required for Slit2N to inhibit this activation. These data suggest a role for Robo4 in the Slit2N-modulated inhibition of VEGF-C-mediated effects in lymphatic endothelium.

### Robo4 is required for Slit2N to inhibit VEGF-C-induced PI3K/Akt activity in L-LECs

**Figure 6:**
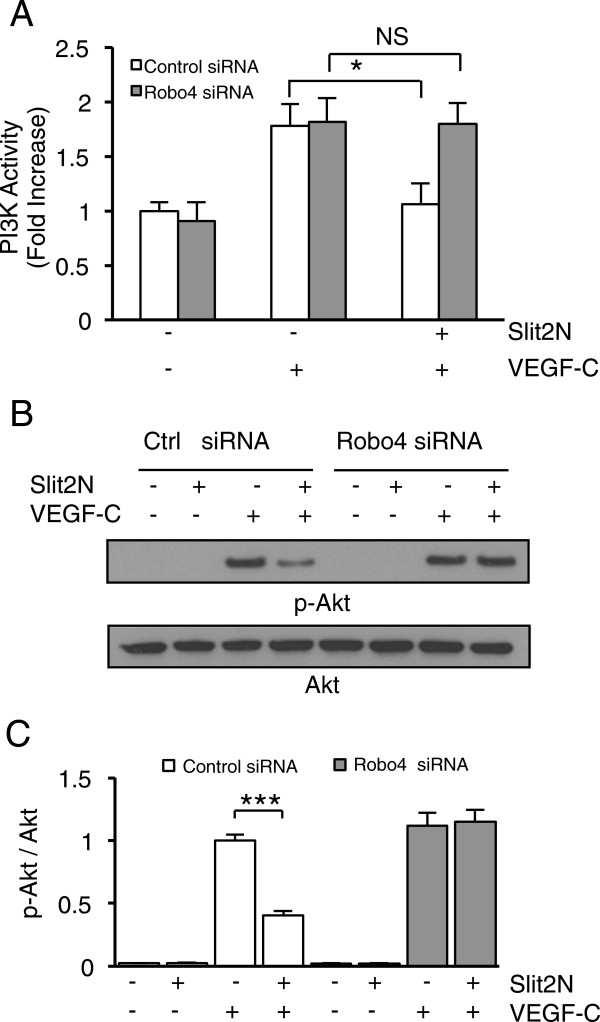
**Slit2N inhibition of VEGF-C-enhanced PI3K activity and Akt phosphorylation in L-LECs is Robo4-dependent. (A)** PI3K activity by ELISA in L-LECs transfected with control siRNAs or Robo4-specific siRNAs, incubated with control or VEGF-C [100 ng/ml]; or after pretreatment with 10 nM Slit2N, then VEGF-C. Data represent the mean ± SD of 3 independent experiments (*p < 0.05; NS: not statistically significant). **(B)** Representative Western blot analysis of Akt phosphorylation in L-LECs transiently transfected with control siRNAs or Robo4-specific siRNAs, and subsequently incubated with 10 nM Slit2N, VEGF-C [100 ng/ml]; or after pretreatment with Slit2N, then VEGF-C. Total Akt used as loading control. **(C)** Quantitative analysis of Akt phosphorylation in L-LECs transiently transfected with control siRNAs or Robo4-specific siRNAs, and subsequently incubated with 10 nM Slit2N, VEGF-C [100 ng/ml]; or after pretreatment with Slit2N, then VEGF-C. Band intensity of each lane from Figure 6B was determined by densitometry. The ratio of p-Akt/total Akt in control siRNA-transfected L-LECs incubated with VEGF-C alone was set to “1” and all other ratios were determined vs. this control. Data represent the mean ± SD of 3 independent experiments (***p < 0.001).

To determine if Slit2N inhibition of VEGF-C-induced PI3K/Akt activation is Robo4-dependent, we used L-LECs transfected with control siRNAs or Robo4-specific siRNAs, as previously described. We set the PI3K activity of untreated control siRNA-transfected cells to “1” and calculated the fold change in activity after incubation with VEGF-C, or after incubation with Slit2N followed by VEGF-C, relative to the untreated controls (Figure 6A). Incubation with VEGF-C enhanced PI3K activity in both sets of transfectants by nearly 100% (Figure 6A). Pretreatment with Slit2N significantly decreased VEGF-C-enhanced PI3K activity in the control siRNA-transfected L-LECs; however, Slit2N had no discernible effect on the cells with reduced Robo4 expression (Figure 6A). These data indicate that Robo4 is required to affect Slit2N-mediated inhibition of VEGF-C-enhanced PI3K activity in L-LECs.

We also examined the role of Robo4 on the Slit2N inhibition of VEGF-C-induced activation of Akt, using the same L-LEC transfectants and conditions described above. We found no Akt activation in either group of untreated transfectants; likewise, Slit2N alone did not induce Akt activation (Figure 6B and C). Incubation with VEGF-C induced similar levels of Akt phosphorylation in both sets of transfectants; however, pretreatment with Slit2N significantly reduced VEGF-C-induced Akt activation only in the transfectants with endogenous Robo4 levels (Figure 6B and C, Control siRNA +/+). Slit2N had no effect on the VEGF-C-induced activation of Akt in the L-LECs with reduced Robo4 levels (Figure 6B and C, Robo4 siRNA +/+). These data indicate that Slit2N can inhibit VEGF-C-induced activation of Akt in L-LECs, and that inhibition by Slit2N is Robo4-dependent.

### Slit2N inhibition of VEGF-C-enhanced growth, migration, and tube formation of L-LECs is Robo4-dependent

**Figure 7:**
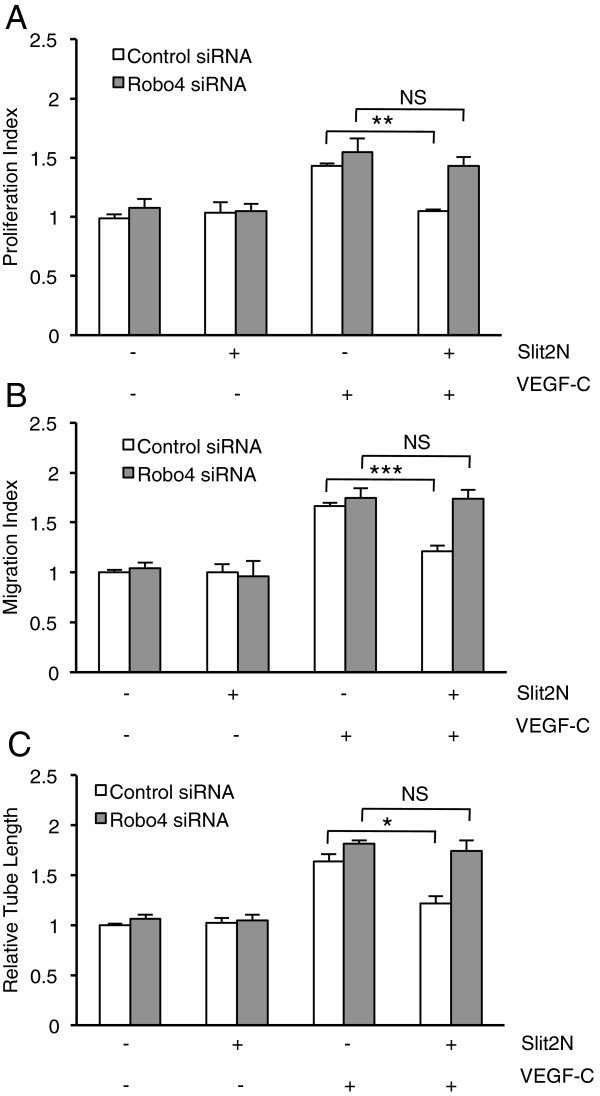
**Slit2N inhibits VEGF-C-enhanced growth, migration, and tube formation of L-LECs in a Robo4-dependent manner. (A)** Proliferation of L-LECs transiently transfected with control siRNAs or Robo4-specific siRNAs as assessed by MTS assay after treatment with control, 10 nM Slit2N, VEGF-C [100 ng/ml]; or after preincubation with Slit2N, then VEGF-C. Data represent the mean ± SD of 3 independent experiments (**p < 0.01; NS: not statistically significant). **(B)** Transwell migration of L-LECs transiently transfected with control siRNAs or Robo4-specific siRNAs after treatment with control, 10 nM Slit2N, VEGF-C [100 ng/ml]; or after preincubation with Slit2N, then VEGF-C. Data represent the mean ± SD of 3 independent experiments (***p < 0.001; NS: not statistically significant). **(C)** Relative length of tubes formed by L-LECs transiently transfected with control siRNAs or Robo4-specific siRNAs as assessed by *in vitro* tube formation assay on ECM after treatment with control, 10 nM Slit2N, VEGF-C [100 ng/ml]; or after preincubation with Slit2N, then VEGF-C. Data represent the mean ± SD of 3 independent experiments (*p < 0.05; NS: not statistically significant). For panels **A**, **B**, and **C**, proliferative index, migration index, and relative tube length, respectively, were set to “1” for control-siRNA-transfected, untreated cells. Data for all other conditions were calculated relative to these controls.

Finally, we queried if Robo4 was required for Slit2N to inhibit the proliferation, migration, and tube formation of L-LECs enhanced by VEGF-C. We repeated the functional assays, illustrated in Figure 1A, B, and D, in L-LEC transient transfectants, which expressed control siRNAs or Robo4-specific siRNAs, as previously described. In these assays, we set the average proliferation levels, migration levels, and tube lengths of untreated control siRNA transfectants to “1,” and calculated the fold change after incubation with recombinant Slit2N and/or VEGF-C, relative to the untreated controls (Figure 7). Slit2N alone had no effect on any of these functions in either set of transfectants, but incubation with VEGF-C enhanced proliferation, transwell migration and average tube length significantly (Figure 7A, B, and C, respectively). Moreover, VEGF-C enhanced these activities to a similar extent in both groups of L-LEC transfectants (Figure 7). When we pretreated the cells with Slit2N before incubating them with VEGF-C, there was a significant inhibition of VEGF-C-enhanced proliferation, migration, and tube length of the L-LEC transfectants with endogenous Robo4 expression (Control siRNA, +/+, Figure 7A, B, and C, respectively); however, Slit2N had no significant effect on these VEGF-C-enhanced activities in the L-LEC transfectants with diminished Robo4 levels (Robo4 siRNA, +/+, Figure 7A, B, and C). These data indicate that sufficient levels of Robo4 appear to be necessary for Slit2N to inhibit these VEGF-C-enhanced functions.

## Discussion

While earlier studies revealed a role for Slit2 and Robo4 in modulating vascular endothelial functions [52-54], there is limited information on the effects of this ligand and receptor in lymphatic endothelium. We found that VEGF-C enhanced the proliferation, migration, and tube formation of L-LECs (Figure 1). These *in vitro* functions have *in vivo* correlates that are critical components of lymphangiogenesis [67]. Slit2N inhibited these enhanced functions (Figure 1) by modulating signaling through VEGF-C and its cognate receptor, VEGFR-3 (Figure 2). Our results provide a mechanism to elucidate previous results, e.g., that Slit2 treatment of cells of endothelial and epithelial origin inhibited their migration and proliferation *in vitro*[68-71], and tumor growth and metastasis *in vivo*[72,73].

In contrast, a prior study by Yang et al. concluded that Slit2 enhanced the *in vitro* tube formation of dermal HMVECs [56]; however, skin-derived endothelial cells and lung-derived endothelial cells have different Robo expression profiles (Figure 5A). HMVECs express high levels of Robo1 and low Robo4. The L-LECs used in our study express high levels of Robo4 (Figure 5A). This suggests that pro- or anti-lymphangiogenic effects of Slit2 may be modulated by multiple factors including the tissue of origin and the Robo receptor with which Slit2 interacts.

Receptor tyrosine kinases (RTKs), including the VEGF receptors, typically are activated by their cognate ligands and modulated by a variety of biological processes including dimerization, internalization, degradation, and receptor presentation [2,61,67,74,75]. Internalization and activation of both VEGFR-2 and VEGFR-3 can trigger downstream signaling through the MAP kinases ERK1/2 and PI3K/Akt in vascular endothelial cells [59,76]. We provide new evidence that pretreatment with Slit2N can inhibit VEGF-C-induced PI3K/Akt signaling in L-LECs (Figure 4C, D, and E), thereby modulating VEGFR-3 presentation levels on the cell surface (Figure 3D and E).

Although both Slit2N and VEGF-C alone induced the internalization of VEGFR-3 (Figure 3A, B, D, and E), only VEGF-C decreased total VEGFR-3 levels, and Slit2N pretreatment did not alter these VEGF-C-induced effects on VEGFR-3 (Figure 3F). These data suggest that Slit2N and VEGF-C may decrease VEGFR-3 surface expression by different mechanisms. One potential explanation is that Slit2N alone may induce the endocytosis of VEGFR-3 into clathrin-mediated endosomes, which traffic back to the cell membrane and facilitate the recycling of VEGFR-3 on the cell surface [2]. The increase in surface VEGFR-3 between 15 and 30 minutes post-incubation with Slit2N is consistent with this hypothesis (Figure 3A and B). After incubating with VEGF-C for 15 minutes, surface expression of VEGFR-3 decreased by half and remained at that level 30 minutes post-incubation; however, after 30 minutes, total VEGFR-3 levels also decreased (Figure 3D, E, and F). These data suggest that VEGF-C does not induce the recycling of VEGFR-3 to the cell surface; rather, they suggest that VEGF-C may target VEGFR-3 to the lysosomes for degradation.

Our work and that of others have shown that Slit2 affects cytoskeletal reorganization, largely by regulating actin polymerization, and by modulating cytoskeletal signaling pathways and the association of key cytoskeletal proteins, including actin [77], WASp [77,78], LSP1 [77], Arp2/3 [77,78], mDia2 (our unpublished data), Fli1 (our unpublished data), paxillin [52,79] and Arf6 [52,79]. Of note, the latter two proteins are important molecules for endocytosis, the key biological process involved in receptor internalization and cell surface presentation [52,80]. By modulating the cytoskeleton, Slit2 has been shown to enhance vascular stability [52,79], inhibit HIV-induced migration of dendritic cells [77], inhibit the infection of CD4^+^ T-cells by HIV-1 [81], block cell-to-cell transmission of HIV-1 (our unpublished data), and inhibit PDGF-induced migration of smooth muscle cells [78]. Therefore, we hypothesize that Slit2N induces the internalization of VEGFR-3 by modulating the L-LEC cytoskeleton. Additional experiments are needed to elucidate the specific cytoskeletal proteins affected by Slit2N that induce VEGFR-3 internalization in these cells.

L-LECs secrete Slit2 and express Robo1 and Robo4 ([82] and Figure 5A). Currently, there are two hypotheses by which Slit2 may signal through Robo4. One suggests that Slit2 binds directly to Robo4 to initiate downstream signaling [48,66]. The other proposes that Slit2 binds to Robo1, which then transactivates Robo4 to initiate signaling [83]. We demonstrated that Slit2N inhibited VEGF-C signaling in cells manipulated to express Robo4, but not in corresponding controls that did not express Robo 4 (Figure 5C and D). Similarly, Slit2N inhibited VEGF-C-induced signaling in L-LECs which expressed moderate levels of Robo4, but not in L-LECs which expressed significantly reduced levels of Robo4 (Figure 5E, F, and G). Taken together, these data, and the robust expression of Robo4 on endothelial cells, suggest that the inhibitory effect of Slit2N on VEGF-C-induced signaling in L-LECs is predominantly through Robo4; however, L-LECs also express Robo1, albeit at much lower levels (Figure 5A).

To assess the potential contribution of Robo1 to the inhibitory effects of Slit2N/Robo4 in our study, we transfected L-LECs with control siRNAs or with Robo1-specific siRNAs, and repeated the functional assays illustrated in Figure 7. Incubation with VEGF-C enhanced proliferation, transwell migration and average tube length significantly, and Slit2N inhibited these VEGF-C enhanced activities in both sets of L-LEC transfectants (Additional file 4A, B, and C). These data indicate that Robo1 is not required for inhibition by Slit2N, and it does not contribute to Slit2N/Robo4 signaling in the lung lymphatic endothelial cells used in this study.

## Conclusions

Our data demonstrate a novel role for Slit2N and Robo4 in the inhibition of functions critical for lymphangiogenesis and lymphatic tumor metastasis, including L-LEC proliferation, migration, and tube formation. This study supports continued characterization of these novel, lymphatic modulators and their potential therapeutic applications for the treatment of pathologies associated with lymphatic endothelial dysfunction.

## Methods

### Cells

Primary human L-LECs and primary dermal HMVECs were purchased from Lonza, Inc. (Allendale, NJ), cultured in EBM-2 plus EGM-2MV SingleQuots (Lonza, Inc.) at 37°C and 5% CO_2_, and used between passages 4 and 6 for experiments described herein. 293/VEGFR-3 cells (Genentech, Inc., San Francisco, CA) were cultured in DMEM, 10% FBS at 37°C and 5% CO_2_.

### Reagents

VEGF-C and Slit2N were purchased from ProSpec (East Brunswick, NJ) and PreproTech, Inc. (Rocky Hill, NJ), respectively. Antibodies used include anti-VEGFR-3, anti-p-Tyr, anti-GAPDH, and anti-p-ERK1/2 (Santa Cruz Biotechnology, Santa Cruz, CA); anti-p-Akt (Ser-473), anti-VEGFR-2 (55B11), anti-p-VEGFR-2 (Tyr1175), anti-ERK1/2 (9102), and anti-Akt (4685) (Cell Signaling Technology, Beverly, MA); anti-PI3K p85 (Upstate Biotechnology, Waltham, MA), anti-Robo1 (ab7279), and anti-Robo4 (ab10547) (Abcam Inc., Cambridge, MA).

### Robo4 expression plasmid transfections and Robo4 expression evaluation

pCMV6-AC-RFP and pCMV6-AC-RFP-Robo4 plasmids [82], were transfected into 293/VEGFR-3 cells using SuperFect® Transfection Reagent per manufacturer’s instructions (Qiagen Inc. - USA, Valencia, CA). Robo4 protein levels were determined by Western blot analysis 24 hours after transfection, as previously described [82].

### Construction of the Slit2N adenoviral expression plasmid, viral packaging, transduction, and Slit2N expression evaluation

The ViraPower™ Adenoviral Expression System (Life Technologies, Woburn, MA), which included 293A packaging cells, was used to express Slit2N per manufacturer’s instructions.

To construct the Slit2N ENTRY clone, the Slit2N gene was amplified from pCMV-ENTRY-Slit2N (OriGene Technologies, Inc., Rockville, MD) using the following primers: 5′- CAC CAT GCG CGG CGT TGG CTG GCA GAT GC and 5′- GGG ACC ATG GGT GGA GAA AAC TC. The PCR product was then cloned into the pENTR™/D-TOPO® vector.

The Slit2N expression clone in which Slit2N is fused to a C-terminal V5 tag was generated by performing the LR reaction between pENTR/D-TOPO-Slit2N and pAD/CMV/V5-DEST (Life Technologies) per manufacturer’s instructions. The expression construct was cut with PAC1 and transfected into 293A cells to produce the adenoviral stock. The adenovirus was transduced into L-LECs, and expression levels of Slit2N confirmed after 24 to 48 hours by Western blot analysis, as previously described [82], using a V5 antibody (Life Technologies).

### siRNA transfections and Robo expression evaluation

Robo1- and Robo4-specific and control siRNAs (Santa Cruz Biotechnology, Inc.) were transfected into cells using HiPerFect® Transfection Reagent per manufacturer’s instructions (Qiagen, Inc). Robo protein expression in each group of transfectants was determined by Western blot analysis, 24 hours post-transfection. *Cell proliferation assay –* CellTiter 96® AQ_ueous_ One Solution Cell Proliferation Assay (MTS) was purchased from Promega Corp. (Madison, WI). Twenty-four hours after receiving fresh media, L-LECs (80% confluent) were trypsinized and washed twice with PBS. Cell suspensions of 2 × 10^5^ cells/ml were incubated with a control or 10 nM Slit2N in starvation media (0.5% BSA/DMEM) for 1 hour. Subsequently, 50 μl of each was seeded into 96-well plates, and 50 μl of starvation medium with/without VEGF-C [100 ng/ml] was added to the wells before incubating the cells at 37°C. After 24 hours, 20 μl of CellTiter 96® AQ_ueous_ One Solution Cell Proliferation reagent was added to each well, and incubated at 37°C for 2 hours. Data were collected at OD490.

### Cell migration assay

L-LEC migration assay was performed using 24-well transwell permeable supports with 6.5 μm pore filters (Corning, Inc., Lowell, MA). The undersides of the filters were coated with 25 ng/ml fibronectin. Twenty-four hours after receiving fresh media, L-LECs were trypsinized, rinsed twice with PBS, resuspended to 1 × 10^5^ cells/ml, and pretreated with 10 nM Slit2N. After 1 hour, 100 μl of the cell suspension was added to the top chamber of each transwell. Starvation media (0.5% BSA/DMEM) (600 μl) with/without VEGF-C [100 ng/ml] was added to each lower chamber. The cells were incubated at 37°C, 5% CO_2_, for 4 hours. Non-migrated cells were removed from the upper chambers by swiping the upper surface with a cotton swab. Migrated cells on the underside were fixed and stained with Diff-Quik® stain kit (Siemens Healthcare Diagnostics, Inc., Newark, DE). Eight high-power fields per filter were counted. Migration index was calculated as the average number of migrated cells incubated with Slit2N, VEGF-C, or both vs. the average number of migrated cells incubated with neither Slit2N nor VEGF-C.

### Tube formation assay

L-LEC tube formation was assessed with the *In Vitro* Angiogenesis Assay Kit (ECM625) (Millipore, Corp., Bedford, MA) per manufacturer’s instructions. Briefly, plates were coated with ECMatrix™, artificial extracellular matrix (ECM), and incubated for at least 1 hour at 37°C, 5% CO_2_. Twenty-four hours after receiving fresh media, L-LECs were trypsinized and washed twice with PBS. Cell suspensions of 2 × 10^5^ cells/ml were preincubated with PBS or 10 nM Slit2N in 0.5% BSA/DMEM for 1 hour. Subsequently, 50 μl of each was seeded onto the ECM-coated plates, then 50 μl 0.5% BSA/DMEM with/without VEGF-C [100 ng/ml] was added to the wells before incubating the cells at 37°C overnight. Capillary tube formation images were captured with a digital microscope camera system (100×) in 5 randomly selected fields in each of the wells. “UTHSCSA ImageTool” software (Dept. of Dental Diagnostic Science, University of Texas Health Science Center, San Antonio, TX) was used to quantify the length of tubes per field. Data represent the average tube length ± SD, 3 wells per condition, 5 fields per well. To analyze the effect of Slit2N transduction on VEGF-C-induced tube formation, L-LECs were transduced with control or Slit2N adenovirus. Twenty-four hours after transduction, the cells were seeded onto the ECM-coated plates with/without VEGF-C [100 ng/ml], and incubated at 37°C overnight.

### Slit2N and VEGF-C final concentrations and incubation times

For all signaling and receptor internalization studies, Slit2N was used at [10 nM] and VEGF-C was used at [100 ng/ml], unless otherwise stated. In these studies, L-LECs were incubated with Slit2N for 1 hour and with VEGF-C for 15 minutes, unless otherwise stated.

### PI3K activity

PI3K activity was assayed with the PI3-Kinase Activity ELISA: Pico (Echelon Biosciences, Inc., Salt Lake City, UT). Twenty-four hours after receiving fresh media, L-LECs were incubated in 0.5% BSA/DMEM and 0-10 nM Slit2N for 1 hour, then incubated with/without VEGF-C [100 ng/ml] for 15 minutes. The cells were rinsed, lysed and centrifuged per manufacturer’s instructions. The supernatants were collected and incubated with an anti-PI3K p85 antibody for 1 hour at 4°C. A 50% slurry of Protein A-agarose beads/PBS (60 μl) was added to each tube, and gently rocked for 1 hour at 4°C. The beads were centrifuged and washed to isolate PI3K. PI3K activity was assayed with a kinetic microplate reader (Molecular Devices, Sunnyvale, CA), and absorbance read at 450 nm.

### Immunoprecipitation and Western blot analysis

L-LECs or 293/VEGFR-3 were incubated with PBS or various concentrations of Slit2N for 1 hour, and then stimulated with VEGF-C [100 ng/ml] or PBS as indicated. Cells were washed with PBS and lysed with RIPA buffer plus a protease inhibitor cocktail (cat. #5871, Cell Signaling). Lysates were incubated on ice for 30 minutes before being centrifuged at 13,000 g for 30 minutes. Immunoprecipitation and Western blot analysis were performed as previously described [84]. To quantitate protein from Western blot analyses, blots were scanned, and band intensity measured using ImageJ software (NIH, Bethesda, MD). For each blot analyzed, one condition was identified as the control as stated in figure legends, and its value established as “1.” All other values were calculated relative to this control.

### VEGFR-3 internalization assay

Cell surface biotinylation was performed in a 100 mm dish. Twenty four hours after receiving fresh media, L-LECs were incubated in 0.5% BSA/DMEM and 10 nM Slit2N alone for 15 and 30 minutes, with VEGF-C alone [100 ng/ml] for 15 and 30 minutes, or incubated with 10 nM Slit2N for 1 hour before incubation with VEGF-C [100 ng/ml] for 15 and 30 minutes. Subsequently, surface receptors were labeled with Sulfo-NHS-LC-Biotin (Thermo Fisher Scientific, Waltham, MA) per manufacturer’s instructions. After quenching excess biotin with 100 mM quenching buffer, the cells were washed with TBS, and dissolved in 0.8 ml RIPA buffer to create whole cell lysates. To measure cell surface VEGFR-3, a fraction of the lysates was precipitated with streptavidin agarose beads (Life Technologies). The beads were washed, and proteins were extracted by boiling with sample buffer before Western blot analysis. The total VEGFR-3 expression was analyzed using the remaining whole cell lysates.

### Statistical analysis

Statistical analyses were performed using a standard two-tailed Student’s T-test. p-values less than 0.05 were considered statistically significant.

## Abbreviations

Robo4: Roundabout-4; VEGF: Vascular endothelial growth factor; VEGFR: VEGF receptor; Slit2N: N-terminal fragment of Slit2; L-LEC: Lung-derived lymphatic endothelial cell; PI3K: Phosphatidylinositide 3-kinase; Akt: Protein kinase B; LRR: Leucine-rich repeat; Fn: Fibronectin; CC: Cytoplasmic conserved; Slit2C: C-terminal fragment of Slit2; HMVEC: Human microvascular endothelial cell; HUVEC: Human umbilical vein endothelial cell; ECM: Extracellular matrix.

## Competing interests

The authors declare that they have no competing interests.

## Authors’ contributions

Conceived and designed the experiments: JEG JY XZ PMK DYL. Performed the experiments: JY SJ. Analyzed the data: JEG JY XZ PMK SJ DYL. Contributed reagents/materials/analysis tools: DYL. Created the recombinant Slit2N protein: WZ. Wrote the manuscript: JEG JY PMK XZ. Critically read the manuscript: JEG PMK XZ DYL. Revised the manuscript: JEG PMK. All authors read and approved the final manuscript.

## Supplementary Material

Additional file 1 **Slit2N has no effect on the association of VEGFR-3 with VEGFR-2 or with Alpha 5 integrin.** VEGFR-3 immunoprecipitation and Western blot analysis of VEGFR-2 and Alpha 5 integrin, with and without Slit2N incubation, in L-LECs. VEGFR-3 used as loading control. Click here for file

Additional file 2 **Slit2N has no effect on the activation of ERK1/2 in L-LECs.** Western blot analysis of phosphorylated ERK1/2 in L-LECs incubated for various times with Slit2N [10 nM]. Total ERK1/2 used as loading control. Click here for file

Additional file 3 **Slit2N has no effect on the activation of Akt in L-LECs.** Western blot analysis of phosphorylated Akt in L-LECs incubated for various times with Slit2N [10 nM]. Total Akt used as loading control. Click here for file

Additional file 4 **Robo1 is not required for Slit2N to inhibit VEGF-C-enhanced growth, migration, and tube formation of L-LECs.** (A) Proliferation of L-LECs transiently transfected with control siRNAs or Robo1-specific siRNAs as assessed by MTS assay after treatment with control, 10 nM Slit2N, VEGF-C [100 ng/ml]; or after preincubation with Slit2N, then VEGF-C. Data represent the mean ± SD of 3 independent experiments (**p < 0.01; ***p < 0.001). (B) Transwell migration of L-LECs transiently transfected with control siRNAs or Robo1-specific siRNAs after treatment with control, 10 nM Slit2N, VEGF-C [100 ng/ml]; or after preincubation with Slit2N, then VEGF-C. Data represent the mean ± SD of 3 independent experiments (**p < 0.01; ***p < 0.001). (C) Relative length of tubes formed by L-LECs transiently transfected with control siRNAs or Robo1-specific siRNAs as assessed by *in vitro* tube formation assay on ECM after treatment with control, 10 nM Slit2N, VEGF-C [100 ng/ml]; or after preincubation with Slit2N, then VEGF-C. Data represent the mean ± SD of 3 independent experiments (***p < 0.001). For panels A, B, and C, proliferative index, migration index, and relative tube length, respectively, were set to “1” for control-siRNA-transfected, untreated cells. Data for all other conditions were calculated relative to these controls. Click here for file
