# Supplementary material for: Slit2N and Robo4 regulate lymphangiogenesis through the VEGF-C/VEGFR-3 pathway
Source: Cell Commun Signal. 2014 Apr 7;12:25. doi: 10.1186/1478-811X-12-25 (PMC4122147; doi:10.1186/1478-811X-12-25)
Supplement: Additional file 1 — Slit2N has no effect on the association of VEGFR-3 with VEGFR-2 or with Alpha 5 integrin. VEGFR-3 immunoprecipitation and Western blot analysis of VEGFR-2 and Alpha 5 integrin, with and without Slit2N incubation, in L-LECs. VEGFR-3 used as loading control. [file 1478-811X-12-25-S1.PDF]

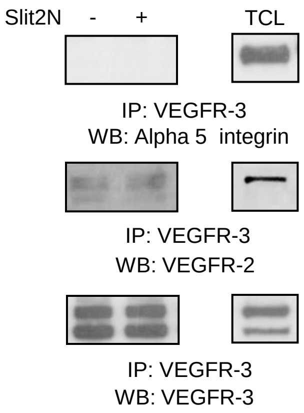

Additional file 1: Slit2N has no effect on the association of VEGFR-3 with VEGFR-2 or with Alpha 5 integrin. VEGFR-3 immunoprecipitation and Western blot analysis of VEGFR-2 and Alpha 5 integrin, with and without Slit2N incubation, in L-LECs. VEGFR-3 used as loading control.
