# Supplementary material for: Slit2N and Robo4 regulate lymphangiogenesis through the VEGF-C/VEGFR-3 pathway
Source: Cell Commun Signal. 2014 Apr 7;12:25. doi: 10.1186/1478-811X-12-25 (PMC4122147; doi:10.1186/1478-811X-12-25)
Supplement: Additional file 3 — Slit2N has no effect on the activation of Akt in L-LECs. Western blot analysis of phosphorylated Akt in L-LECs incubated for various times with Slit2N [10 nM]. Total Akt used as loading control. [file 1478-811X-12-25-S3.PDF]

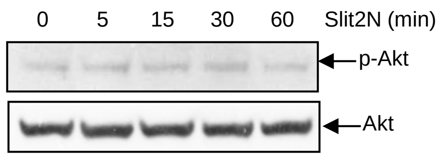

Additional file 3: Slit2N has no effect on the activation of Akt in L-LECs. Western blot analysis of phosphorylated Akt in L-LECs incubated for various times with Slit2N [10nM]. Total Akt used as loading control.
