# Supplementary material for: Slit2N and Robo4 regulate lymphangiogenesis through the VEGF-C/VEGFR-3 pathway
Source: Cell Commun Signal. 2014 Apr 7;12:25. doi: 10.1186/1478-811X-12-25 (PMC4122147; doi:10.1186/1478-811X-12-25)
Supplement: Additional file 4 — Robo1 is not required for Slit2N to inhibit VEGF-C-enhanced growth, migration, and tube formation of L-LECs. (A) Proliferation of L-LECs transiently transfected with control siRNAs or Robo1-specific siRNAs as assessed by MTS assay after treatment with control, 10 nM Slit2N, VEGF-C [100 ng/ml]; or after preincubation with Slit2N, then VEGF-C. Data represent the mean ± SD of 3 independent experiments (**p < 0.01; ***p < 0.001). (B) Transwell migration of L-LECs transiently transfected with control siRNAs or Robo1-specific siRNAs after treatment with control, 10 nM Slit2N, VEGF-C [100 ng/ml]; or after preincubation with Slit2N, then VEGF-C. Data represent the mean ± SD of 3 independent experiments (**p < 0.01; ***p < 0.001). (C) Relative length of tubes formed by L-LECs transiently transfected with control siRNAs or Robo1-specific siRNAs as assessed by in vitro tube formation assay on ECM after treatment with control, 10 nM Slit2N, VEGF-C [100 ng/ml]; or after preincubation with Slit2N, then VEGF-C. Data represent the mean ± SD of 3 independent experiments (***p < 0.001). For panels A, B, and C, proliferative index, migration index, and relative tube length, respectively, were set to “1” for control-siRNA-transfected, untreated cells. Data for all other conditions were calculated relative to these controls. [file 1478-811X-12-25-S4.PDF]

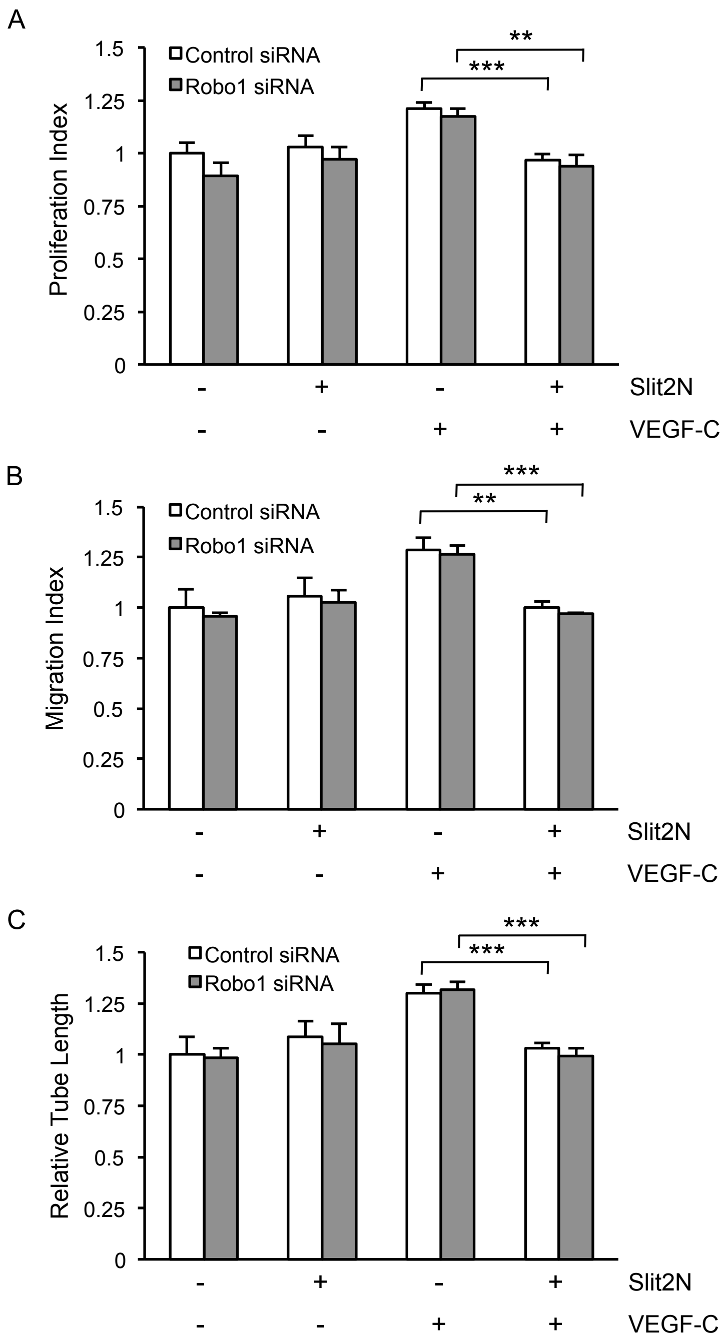

Additional file 4. Robo1 is not required for Slit2N to inhibit VEGF-C-enhanced growth, migration, and tube formation of L-LECs. (**A**) Proliferation of L-LECs transiently transfected with control siRNAs or Robo1-specific siRNAs as assessed by MTS assay after treatment with control, 10nM Slit2N, VEGF-C [100ng/ml]; or after preincubation with Slit2N, then VEGF-C. Data represent the mean ± SD of 3 independent experiments (**p < 0.01; ***p < 0.001). (**B**) Transwell migration of L-LECs transiently transfected with control siRNAs or Robo1-specific siRNAs after treatment with control, 10nM Slit2N, VEGF-C [100ng/ml]; or after preincubation with Slit2N, then VEGF-C. Data represent the mean ± SD of 3 independent experiments (**p < 0.01; ***p < 0.001). (**C**) Relative length of tubes formed by L-LECs transiently transfected with control siRNAs or Robo1-specific siRNAs as assessed by *in vitro* tube formation assay on ECM after treatment with control, 10nM Slit2N, VEGF-C [100ng/ml]; or after preincubation with Slit2N, then VEGF-C. Data represent the mean ± SD of 3 independent experiments (***p < 0.001). For panels A, B, and C, proliferative index, migration index, and relative tube length, respectively, were set to “1” for control-siRNA-transfected, untreated cells. Data for all other conditions were calculated relative to these controls.
